# Optically Addressing Exciton Spin and Pseudospin in Nanomaterials for Spintronics Applications

**DOI:** 10.1021/acsaom.3c00299

**Published:** 2023-11-16

**Authors:** Daphné Lubert-Perquel, Swagata Acharya, Justin C. Johnson

**Affiliations:** Materials, Chemical, and Computational Science Directorate, National Renewable Energy Laboratory, 15013 Denver West Parkway, Golden, Colorado 80401, United States

**Keywords:** exciton, coherence, spin-valley, ultrafast, spin-polarization, 2D materials, 2D magnets

## Abstract

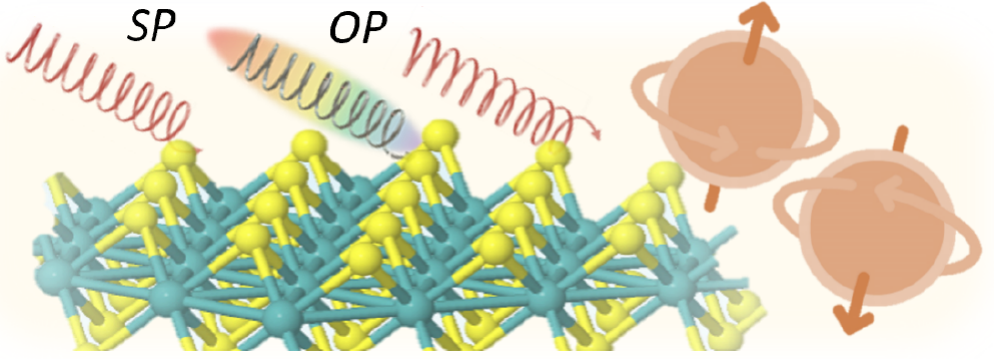

Oriented exciton spins that can be generated and manipulated optically are of interest for a range of applications, including spintronics, quantum information science, and neuromorphic computing architectures. Although materials that host such excitons often lack practical coherence times for use on their own, strategic transduction of the magnetic information across interfaces can combine fast modulation with longer-term storage and readout. Several nanostructure systems have been put forward due to their interesting magneto-optical properties and their possible manipulation using circularly polarized light. These material systems are presented here, namely two-dimensional (2D) systems due to the unique spin-valley coupling properties and quantum dots for their exciton fine structure. 2D magnets are also discussed for their anisotropic spin behavior and extensive 2D magnetic states that are not yet fully understood but could pave the way for emergent techniques of magnetic control. This review also details the experimental and theoretical tools to measure and understand these systems along with a discussion on the progress of optical manipulation of spins and magnetic order transitions.

## 1 Introduction

**Figure 1:**
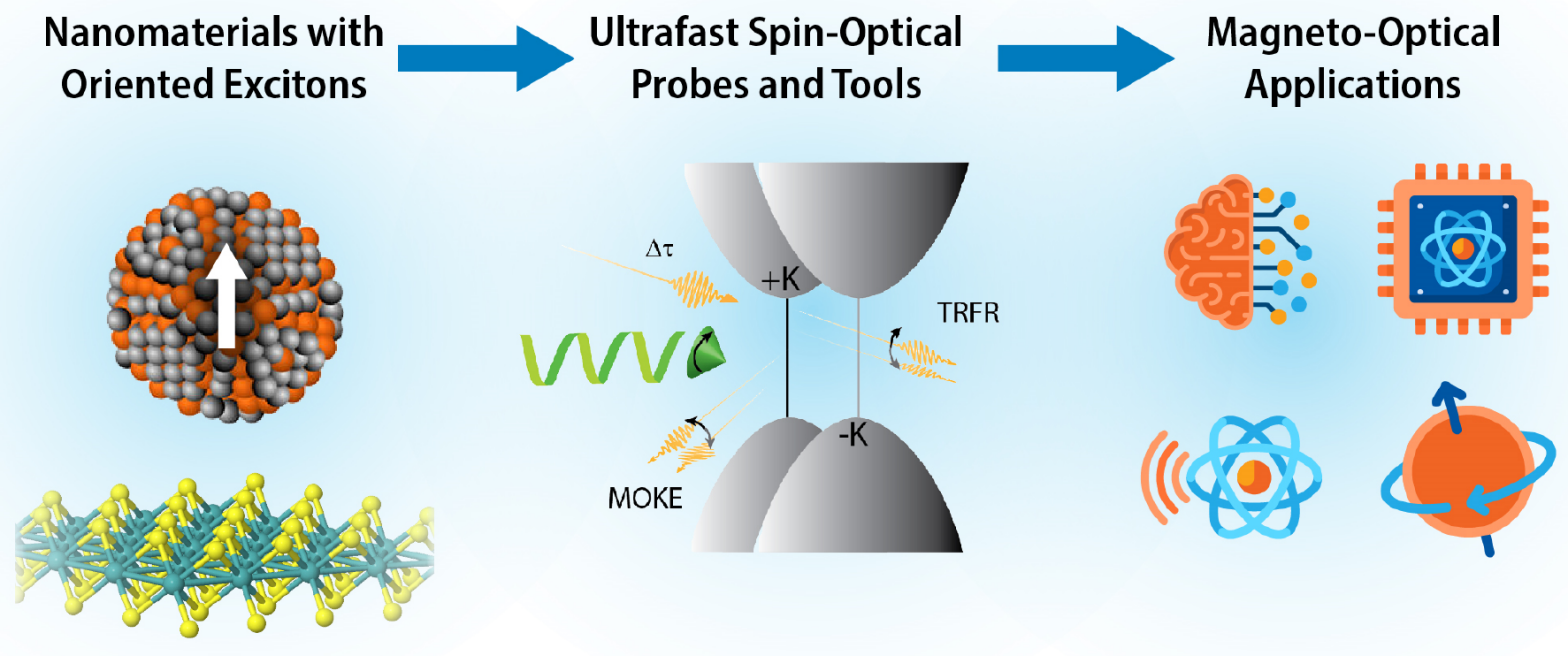
Outline of the review, from materials properties to processes and probes to applications.

Spin or pseudospin degrees of freedom are convenient carriers of digital information that have well-established behavior and can cooperate toward useful phenomena such as controllable magnetic switching. However, in many conventional bulk semiconductors, manipulation and detection of spin states requires extremely large magnetic fields, due to the inherently small DC magnetic susceptibility that often accompanies electron(hole) spin in most materials, reducing the utility of spin-based computation and sensing in practical and energy-efficient environments. Further, the energies of most direct spin transitions typically fall in the radio frequency or microwave regime, for which multiple full cycles of radiation required to manipulate the spin state will put a speed limit on operations that may prevent broad utility. Development of optoelectronic or opto-magnetic effects that obviate the need for large magnetic fields and can act on femtosecond or picosecond time scales is thus highly advantageous and may open new avenues for more versatile spin-based applications (Figure 1). In particular, optical methods of distinguishing and manipulating spins in nanomaterials have several advantages: speed (through ultrafast optical pulses), selectivity (through resonance and selection rules), reduced energy consumption, and scalability. Over the past decade, the physics of such methods has become better understood, and demonstrations of optically tunable spin manipulation and magnetic effects have grown considerably.

Although there are many examples of electron or nuclear spin polarization implemented and manipulated for various purposes (i.e., nuclear magnetic resonance (NMR) and electron spin resonance (ESR) of radicals), we restrict our focus here to excitons as spin carriers, as they are often the primary photoexcited species in nanomaterials. Excitons are also often strongly emissive, enabling a connection between the spin state and the characteristics of photons absorbed or emitted. The emission typically has a narrow line width and separately assignable peaks that are specific to the exciton fine structure (e.g., singlet–triplet splitting). The challenge of creating a well-defined ensemble of spin-polarized states can be addressed with a combination of photon polarization control and the known excitonic properties of a particular nanoscale system. The high degree of electron–hole correlation leads to strong exchange interactions, which can enhance spin-valley mixing and reduce coherence times, but it can also enable spin-state detection and provide clues about the nature of the excitons themselves (e.g., the influence of quantum confinement). One of the most intriguing attributes of nanostructures is the possibility to control electron–hole exchange through size, shape, and defect engineering, including the introduction of heterojunctions, to access a regime where beneficial and deleterious effects are rationally controlled.

In terms of applications, the spintronics field relies upon generation, manipulation, transmission, and accounting of spins. As operations become more complex and larger in scale, fidelity and speed become paramount. Optical methods have distinct advantages over purely electrical or magnetic methods in several of these categories. In fields related to sensing, spin orientation, lifetime, or phase memory are highly dependent on the local chemical environment and can be measured accurately to detect localized interactions between a nanomaterial and its surroundings. Nitrogen-vacancy centers in diamond provide a good example for what optical quantum sensors can achieve based on light-induced spin polarization and its environment-dependent decay.^1^ Recently, “molecular” spin sensors have emerged as tailorable mimics of NV-diamond sensors.^2^ Synonomous with molecules, analogues involving semiconductor nanostructures should be almost infinitely tailorable, providing high surface area objects of designed size and shape. Quantum confinement enables further control, as it tunes the energetic position and splitting of exciton features into convenient regions of the optical spectrum.

In this review we will describe the origin of spin and pseudospin in nanoscale excitonic systems and present demonstrations of their optical detection and manipulation found in prior literature. The salient nanoscale systems are layered two-dimensional (2D) materials and quantum dots/wells, as they possess spin-valley polarization and exciton fine structure, respectively, that can be leveraged for optical generation of spin orientation. Work in 2D materials is particularly emergent in the past half decade and will be featured, with other quantum-confined systems as a counterpoint. Certain 2D materials also exhibit ferromagnetism, and the interaction between excitons and the persistent magnetization is of considerable interest. Once general guidelines about exciton orientation in these materials is established, we turn to describing the optical techniques used to probe and manipulate spin dynamics. Finally, we will look forward to new implementations of optically oriented excitons using these materials and anticipate potential applications.

## 2 Materials Physics and Electronic Structure

This section first introduces the three types of nanoscale materials systems reviewed here as interesting candidates for device applications: 2D transition metal dichalcogenides (TMDCs), 2D magnets, and quantum dots (QDs). The underlying physics for the optical drive and/or probe of spin properties is distinct for each of these classes, and the electronic structure and associated fundamental optoelectronic behavior can be juxtaposed with each other toward distinguishing meritorious vs deleterious properties in the spintronics field. For TMDCs the pseudospin valley degree of freedom and its optical selection rules are exploited for optical manipulation of valley pseudospins. For QDs the crystal structure in the limit of quantum confinement dictates the exciton fine structure, assigned with angular momentum projection (i.e., proxy for spin) character and connected to the ground state by clear selection rules for photon polarization. By contrast, most 2D magnets are strongly correlated systems with excitons localized near magnetic atoms, with the chemical nature of the material providing consequences for how exciton and spin are coupled. Simultaneous with the goal of spin manipulation are the complex exciton dynamics that lead to decoherence and nontrivial competing mechanisms that directly affect the potential for device application.

### 2.1 Transition Metal Dichalcogenides

**Figure 2:**
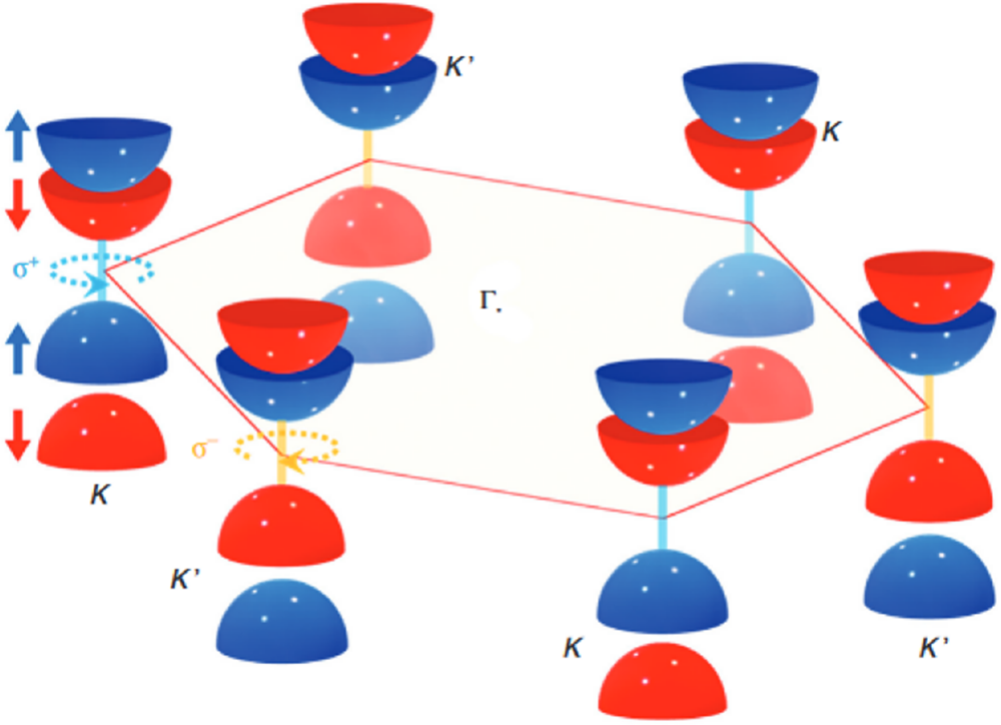
(a) Schematic illustrating the valley-dependent optical selection rules for 2D monolayer tungsten-based TMDCs. Due to strong SOC, the conduction and valence bands are spin-split which results in spin-valley locking. As a result, σ^+^ (σ^–^) circularly polarized light will preferentially populate valleys + K (−K). Blue (red) stands for spin up (down). Adapted with permissions from ref (8). CC BY 4.0 DEED.

In addition to the electron’s charge and spin, valley pseudospin is another binary degree of freedom that can be exploited to store and carry information. The valley is defined as local maximum (minimum) in the valence (conduction) band of a material. Of interest here are hexagonal 2D materials, specifically monolayer or few layer TMDCs. 2D systems are notable for their lack of inversion symmetry combined with time-reversal that result in degenerate and inequivalent valleys at the +K and −K points of the Brillouin zone, resulting in pseudospin behavior. One of the physical quantities used to describe electrons in Bloch bands is the orbital magnetic moment (**m**). At the +K and −K points, the valence band states are comprised of *d*_*x*^2^–*y*^2^_ orbitals with *m* = ± 2 from the transition metal. This results in large spin splitting due to the spin–orbit coupling (SOC) and therefore only the valence states of the top sub-band need to be considered. Note that the spin splitting is larger for W-based TMDCs than for Mo-based TMDCs. This strong interaction results in coupling between the spin-valley components, also known as spin-valley locking, where + K (−K) can only have spin-up (down) for holes that sit on the valence band edge (Figure 2). Conversely, the conduction band states are primarily comprised of d_*z*_^2^ orbitals with *m* = 0 that do not contribute to the SOC. Nonetheless, small splitting of the conduction band does occur due to the *d*_*xz*_, *d*_*yz*_ orbitals of the transition metal and the *p*_*x*_ and *p*_*y*_ orbitals of the chalcogens, with spin splitting of opposite signs for W and Mo based TMDCs.^3^ Moreover, the valley-contrasting **m** leads to valley-dependent optical selection rules i.e.: σ^+^ (σ^–^) circularly polarized light will preferentially populate valleys + K (−K), Figure 2.^4−8^ Initialization of a spin-valley state via helical optical excitation was then demonstrated by Lu et al.^9^ The orbital magnetic moment **m** also allows coupling with magnetic fields, of relevance for spin manipulation.^10,11^ In Section 3, the optical spectroscopy techniques used to investigate spin-valley coupling are discussed.

Spin-valley coupling is significant as it increases the polarization lifetimes and can be manipulated to tune both the spin and valley characteristics.^6,12^ As such, material^13,14^ and surface engineering^15^ have been employed to develop systems with tunable spin-valley coupling. Furthermore, the field has seen a recent push to harness this interaction in a range of applications. Manipulation of the intrinsic magnetization was found to induce spin-valley polarization in a ferromagnetic semiconductor;^16^ manipulation of the spin-valley coupling was demonstrated using a ferroelectric field;^17^ and spin-valley qubits were studied for practical spintronic devices.^18−20^

From bulk to monolayer, several TMDCs are reported to undergo a transition from an indirect bandgap to a direct bandgap semiconductor.^3,21−24^ Owing to this low dimensionality, 2D materials have weak dielectric screening which in turn results in a strong Coulomb interaction. Consequently, these are excitonic systems with large binding energies and oscillator strength.^7,12,25^ Excitons with the electron and hole in the same valley but opposite spin are bright excitons due to the conservation of spin and momentum. The optical signature of TMDCs comes from the splitting of the valence band which results in two resonances commonly labeled as A and B excitons. However, due to the multiple valleys in the band structure of 2D materials, several bright and dark states can be identified. It has been demonstrated that dark excitons could become bright by changing the angle between the detection and polarization planes and more recently by applying an in-plane magnetic field.^26,27^

**Figure 3:**
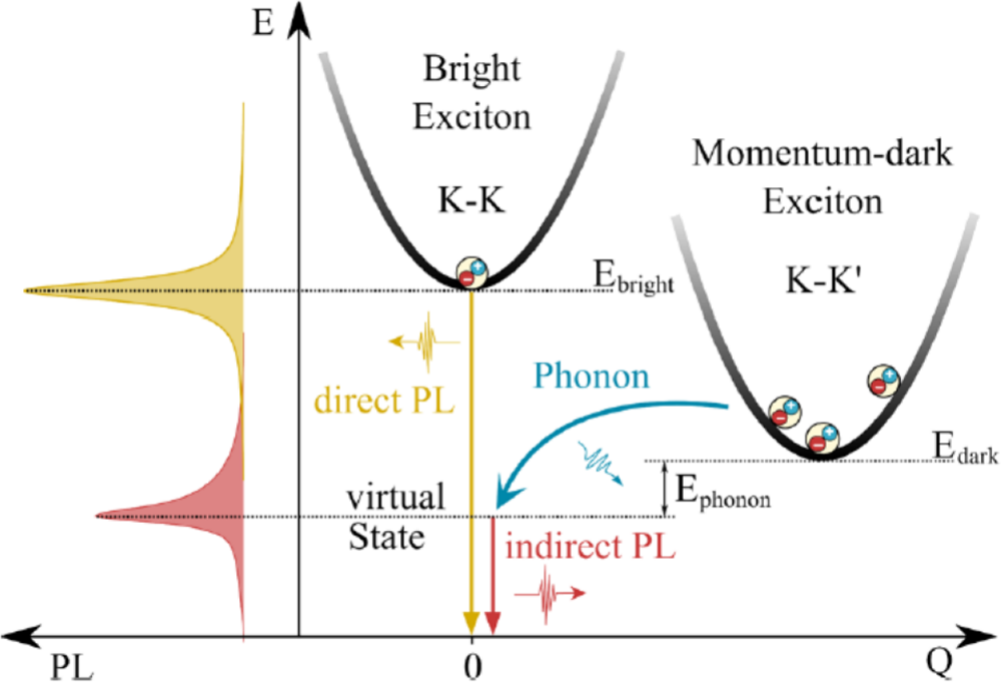
Schematic of excited-state processes in TMDCs. Reproduced with permission from ref (31). Copyright 2022 American Chemical Society.

As we are concerned with the optical manipulation of TMDCs in this review, it is important to understand the multiple processes that occur upon photoexcitation (Figure 3). The population dynamics can be investigated using optical spectroscopy, and each mechanism has a specific optical signature. A recent procedure has been suggested to deconvolute the various contributions, both qualitatively and quantitatively.^28^ The complex excitonic landscape with extensive spin-valley-phonon interactions results in multiple radiative and nonradiative relaxation pathways that need to be considered. Where Mo-based TMDCs have low-lying bright states, in W-based TMDCs, the lowest-lying exciton is momentum dark. These excitons with electrons and holes in different valleys can radiatively recombine via the interaction with a phonon. Experimentally, this appears as an asymmetry in the PL of bright states or as phonon sidebands when the temperature is lowered below 50 K where the bright states are depopulated.^29−31^

Additional nonradiative pathways can include defect-assisted recombination or exciton–exciton annihilation (EEA) in cases with high exciton density. Although not useful for optical manipulation, these pathways need to be considered as they result in important limitations on the efficiency of optoelectronic devices by reducing the exciton lifetime significantly. EEA can be mitigated by h-BN encapsulation, which was found to reduce by 2 orders of magnitude for WS_2_ on SiO_2_ substrates.^32^ In the case of defect-assisted recombination, the logical course would be to reduce the concentration of defects, however this is oftentimes a nontrivial materials challenge.

In addition to population relaxation, loss of coherence of the excitons (i.e., spin-valley polarization memory) can occur due to Coulomb or phonon-mediated scattering. The carrier density or lattice temperature will directly increase the collisional scattering rate, resulting in broadening of the exciton linewidth. The increased linewidth is not only a signature of reduced coherence time but also complicates efforts to detect specific excitonic resonance as peaks become poorly resolved. Local potentials from defects can also lead to inhomogeneous linewidth broadening.^33,34^ Such broadening is equally problematic as homogeneous effects for selecting and measuring excitonic resonances, but the loss of phase memory can typically be reversed through pulsed echo techniques. Moreover, the intervalley electron–hole exchange interaction results in valley decoherence.^35^ Several factors can affect the depolarization dynamics, presenting an opportunity for control of the valley pseudospin. First, an increase in temperature results in increased decoherence reported as a factor 4 between 4 and 125 K.^36^ Then, decoherence can be tuned by strain in TMDC monolayers.^37,38^ Modulation of the valley pseudospin has also been achieved via the Zeeman effect in an external magnetic field^39^ and using a pseudomagnetic field from the optical Stark effect.^40,41^ Similarly, a Zeeman-type polarization applying an external electric field can tune the exciton splitting,^42^ and a polarization reversal of a WSe_2_-based ambipolar transistor was reported.^43^ Finally, due to the optical selection rules, the valley polarization can be controlled by the helicity of light^44^ and by spin-injection.^45^

### 2.2 Two-Dimensional Magnets

**Figure 4:**
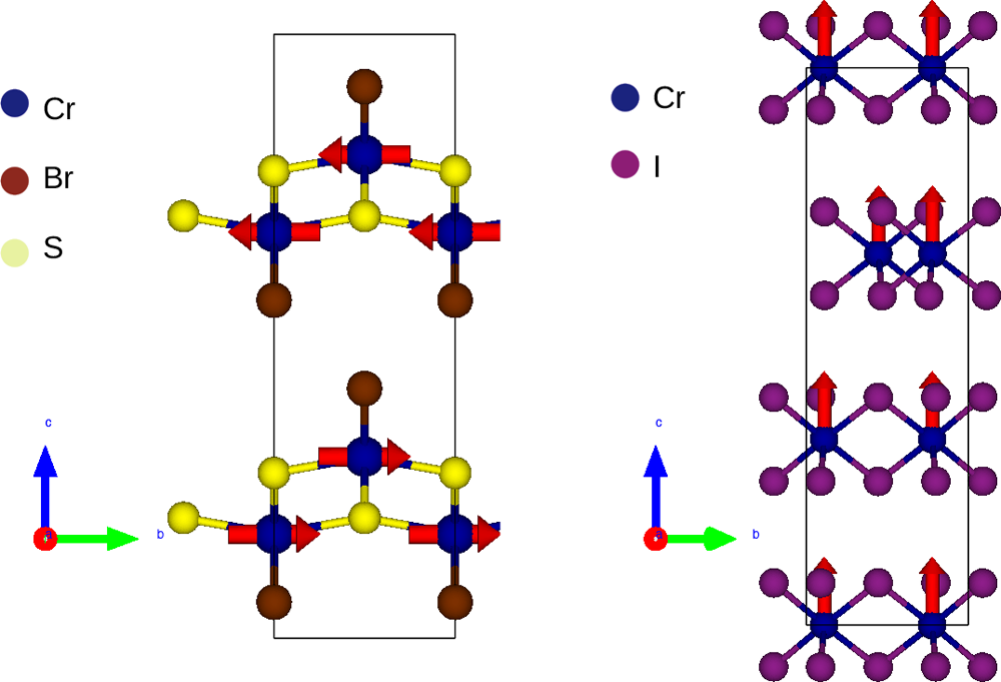
Left, crystal structure of the puckered magnet CrSBr (*a* = 3.504, *b* = 4.738 Å, space group *Pmmn*). Within individual layers the system has ferromagnetic chains of Cr atoms with spins aligning along the *b* axis, while the interlayer coupling is antiferromagnetic. Right, crystal structure of hexagonal magnet CrI_3_ with spins pointing along the *c* axis.

Bulk ferromagnetic Chromium trihalides (see Figure 4) were realized in the 1950s^46^ and 1960s,^47^ but it took nearly 70 years to reduce the system size down to atomically thin two-dimensional ferromagnetic layers.^48,49^ The Mermin-Wagner theorem^50^ dictates that the strong thermal fluctuations at finite temperatures are sufficient to destroy the magnetic order in 2D ferromagnets with isotropic spin interaction. However, over the past few years several ferro- and antiferromagnetic 2D systems have been realized, and this is primarily due to the anisotropic nature of the spin interaction that forms a spin gap and protects the magnetic order from being destroyed by thermal fluctuations.^51,52^ Most ferromagnetic bulk crystals have a magnetic anisotropy and easy-axis of magnetization and when the dimensionality is decreased to a 2D layer, Ising anisotropy is sufficient to maintain the critical temperature. This suggests the magnetic ordering is dominated by magnetic exchange within the 2D layers, and that exchange coupling is weak between layers.

**Figure 5:**
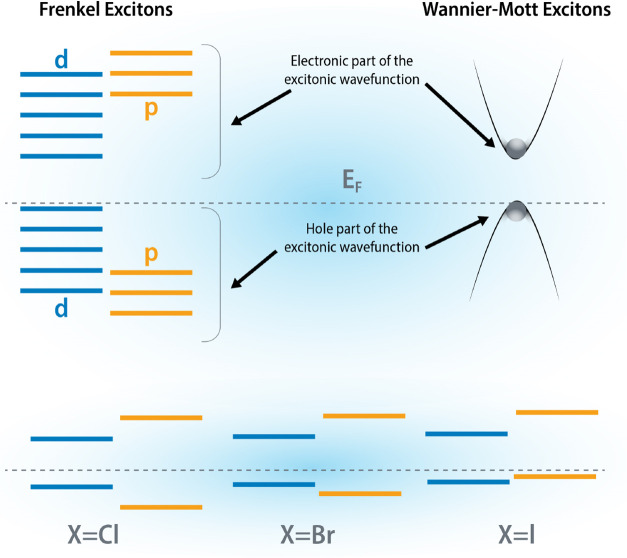
Right, Wannier-Mott excitons, with weak binding energies, are found mostly in *sp* like semiconductors and also in most of the nonmagnetic TMDCs where the hole from the valence band top and electron from the conduction band bottom take part in the exciton formation. In strong contrast, the Frenkel excitons on the left are observed in *pd* and *df* like systems. In 2D correlated magnets, all *p* and *d* states from both valence and conduction bands take part in exciton formation. The hole-*p* states from the heavier ligand (e.g., I from CrI_3_) with more core states (compared to F, Cl, Br) become shallower in the valence (lower portion), leading to two key changes to the essential structure of the excitonic wave function: (i) since the essential *pd* exchange is antiferromagnetic, the nature of the interaction of hole-spin with the overall magnetization of the system (and/or applied magnetic field) can qualitatively be different from those with lighter ligands; (ii) a heavier ligand leads to large spin–orbit mediated changes to electronic and excitonic wave functions. This principle goes beyond the particular material class of CrX_3_, and we should expect changes to the interaction between the exciton spin and applied field primarily mediated by changes in the hole part of the wave function.

While the principles outlined above suggest straightforward behavior, understanding these systems is far from complete. Even within the single layers of chromium trihalides, CrBr_3_^53,54^ and CrI_3_^55^ have the magnetic easy axis perpendicular to the 2D plane, whereas CrCl_3_ has it in-plane. This observation is intriguing for two reasons: (a) the spin–orbit coupling strength enhances in the direction^56^ Cl → Br → I and (b) the valence bands contain more halogen states as the potential of the halogen atom becomes shallower in the direction Cl → Br → I. This makes low energy physics in CrI_3_ largely dominated by the larger spin orbit coupling^56^ of the heavier halogen atoms (Figure 5). However, it is CrCl_3_ with the weakest spin–orbit coupling that exhibits the in-plane magnetic easy axis, a problem that is not fully understood yet.

Our understanding is further limited when it comes to the electronic structure and collective charge excitations in this class of 2D ferromagnets, most of which are strongly correlated in nature, and the electronic and excitonic wave functions are localized within a few angstroms around the magnetic Cr atom.^22,24,56,57^ Ionic ligand-field analysis suggests that in CrX_3_ the Cr *d* states should split into a *t*_2*g*_ triplet and an *e*_*g*_ doublet. Cr^3+^ ions should have a moment of ∼3 μ_B_ with the *t*_2*g*_ majority-spin bands filled, leaving other *d* bands empty. Such qualitative conclusions on the basis of ionic ligand field theory are also confirmed by ab initio density functional theory (DFT) calculations.^58−60^ However, at the quantitative level details start to differ from the fully ionic picture; one important such factor is the degree of hybridization of the *t*_2*g*_ levels with the *p* bands of the ligands, which is controlled by the alignments of *p* and *d* states. This degree of hybridization sensitively depends on the atomic weight of the ligand and the number of core levels, which are shown to be important factors in determining the detailed electronic band structure. Further, when a heavier ligand becomes shallow enough to sit at the top of the valence states close to Fermi energy, spin–orbit coupling plays a crucial role in modifying the electronic and excitonic spectrum of the material. At that point a quantitative understanding of hybridized *pd* manifold becomes crucial for describing reliably both the one- and two-particle properties. The critical role of a high level many-body perturbative approach thus becomes apparent.^56^

The electronic and excitonic wave functions of these systems have been explored recently by applying quasiparticle self-consistent GW theory (QSGW)^61−63^ theory and its higher order diagrammatic extensions. QSGW is a theory that is significantly different from the conventional GW methods in many ways; it modifies the charge density and is determined by a variational principle^64^ and it also contains the entire off-diagonal block of the self-energy matrix, thereby allowing the electronic eigenfunctions to get iteratively updated from its initial starting point. However, to extract the excitonic spectrum we need additional vertex correction to GW. In conventional approaches the optical polarizability is often computed with ladder-vertex corrections by solving a Bethe–Salpeter equation (BSE) starting from a one-shot GW self-energy. In strong contrast to the conventional approaches, in QSGW*^*^65^ the screened coulomb interaction W is computed including ladder-vertex corrections by solving a BSE within Tamm-Dancoff approximation.^66^ Crucially, QSGW*^* methods are fully self-consistent in both one-particle (self-energy Σ and the charge density) and two-particle properties.^67^ This level of the theory incorporates the important physics of how electron–electron Coulomb correlation gets screened when electron–hole bound states form. *G*, Σ, and W*^* are updated iteratively in QSGW*^* until all converge. These results are thus parameter-free and have no starting point bias. Hence, the final result does not depend whether we start from different flavors of DFT or Hartree–Fock theory. For monolayers, single particle calculations (LDA, and energy band calculations with the static quasiparticlized QSGW self-energy Σ^0^(*k*)) were performed on a 12 × 12 × 1 (for bulk, 8 × 8 × 8) *k*-mesh while the (relatively smooth) dynamical self-energy Σ(*k*) was constructed using a 6 × 6 × 1 (for bulk, 4 × 4 × 4) *k*-mesh and Σ^0^(k) extracted from it. The (dominant) RPA part of the polarizability is computed with the tetrahedron method, which helps to facilitate convergence. The size of the two-particle Hamiltonian that we have diagonalized for CrX_3_ 6 × 6 × 24 × 14 (*n*_*k*_× *n*_*k*_ × *N*_*v*_ × *N*_*c*_), i.e., 12 096. For *n*_*k*_ = 9, it is 27 216.

A key observation of QSGW*^* theory is how the electronic wave functions delocalize on the Cr-X bonds compared to DFT: there is a transfer of spectral weight from Cr to X and the bonding becomes more directional, forming one-dimensional chains.^56^ This leads to enhancement of the Cr–Cr coupling mediated through the halogens in QSGW*^* theory, which is underestimated both qualitatively and quantitatively in DFT.

Such quantitative changes that are captured in QSGW*^* theory have important consequences for the exciton wave functions of 2D ferromagnets: i.e., the nature of the coupling of an exciton with the spins of the bath in ferromagnetic systems. It was recently demonstrated^57^ that the coupling between excitons and magnetization is qualitatively different in CrBr_3_ and CrI_3_. Through a combination of the optical spin pumping experiments with the state-of-the-art QSGW*^* theory describing excitonic states in the presence of magnetization, it was concluded that the hole-magnetization coupling has the opposite sign in CrBr_3_ and CrI_3_ and also between the ground and excited exciton state. This is a significant observation considering how similar the structural and magnetic configurations are in these two systems and the fact that there is very little difference between these two materials from the perspective of ligand-field theory. Grzeszczyk et al. revealed such dramatic differences in the exciton-magnetization coupling between the two systems by analyzing the exciton wave functions in band-, orbital-, spin-basis and in real space. The authors also noted that a part of the essential conclusions were different if a nonself-consistent (single-shot) GW theory^22,23^ was used instead of a fully self-consistent theory. A self-consistent, parameter free, many-body perturbative approach appears to be more important in the “Frenkel” limit where the electron and hole parts of the excitonic wave function get delocalized in the band-basis over several valence and conduction bands (Figure 5) (which includes both *p* and *d* characters and contain spins of both kinds), in strong contrast to Wannier-Mott limit where a reasonable description of the electron and hole wave functions from the conduction band bottom and valence band top is sufficient to describe the excitons reliably.

### 2.3 Quantum Dots

Various seminal papers and reviews set the stage for the theory of oriented angular momentum states of quantum dots.^68^ We only summarize the situation here, and direct the reader to more detailed theory for further information. Because purely excitonic states in systems with high spin–orbit coupling have been shown to possess limited coherence times, the field has gravitated toward defects and dopants as the most relevant spin-carrying elements in QDs for QIS applications.^69^ Motivation for studying oriented excitons in QDs remains for fundamental understanding and for applications wherein the optically generated exciton spin orientation can be transduced to other forms (e.g., radical spins or polarized nuclei) in advance of dephasing.

**Figure 6:**
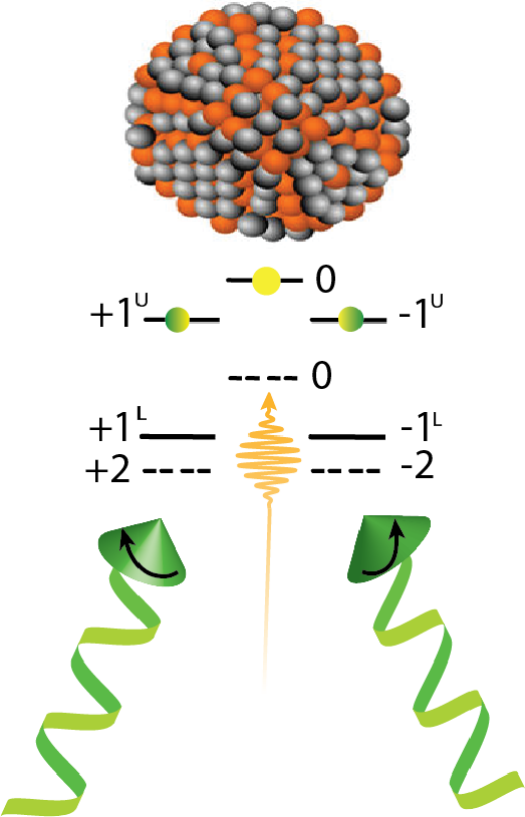
Schematic of the generation of oriented angular momentum states of excitons in spherical quantum dots populated through right and left circularly polarized light excitation. Bright (dark) states are given by bold (dashed) lines. Upper and lower exciton branches are shown, with 0 angular momentum states excited with linear polarization.

We use the commonly encountered case (e.g., CdSe QDs) of spherical semiconductor nanocrystals of hexagonal lattice symmetry and focus on the fine structure of the band edge exciton in an infinite potential (Figure 6).^70^ The 4-fold degenerate hole and 2-fold degenerate electron states produce band-edge exciton states that are split by spin–orbit coupling and crystal field effects. These exciton states are labeled by their angular momentum projections, *F* = 0, ± 1, ± 2. Bright *F* = ± 1 states occur in upper and lower branches and are separated from *F* = ± 2 states, which are formally dark, by the exchange term Δ. In the strong confinement regime, Δ depends on quantum dot size as radius^–3^, though other dependencies can dominate outside of this regime. The splitting of different spin states, dictated by Δ, can be detected through their photoluminescence peak splitting at extremely high magnetic fields,^71^ as the small exchange differences are otherwise difficult to resolve at zero field, especially in the presence of inhomogeneous broadening. However, as we discuss below, measuring transitions between exciton fine structure levels and relating the rate constants to energy splittings based on Fermi’s golden rule provides a zero-field route.

With suitable orientation of the nanocrystal, the *F* = ± 1 states can be populated with circularly polarized light due to the complex (X ± iY) form of the transition dipole moments connecting these states with the ground state. This yields a sample with macroscopically “oriented” excitons in aligned systems, such as lithographically defined QDs or quantum wells. The exponential loss of signal in time is related to the loss of initial spin memory (the phase decoherence), which depends on the type and strength of electron–hole interactions. For quantum dots, exchange and spin–orbit coupling effects, some of which are strongly size dependent, dictate the rate of spin flipping. This has been previously used to determine the extent of electron–hole delocalization and separation in various types of nanostructures.^72,73^ Because the time scales associated with the spin flipping are often picoseconds or faster, the measurement is often considered to be a “snapshot” of the initially delocalized exciton.

Spin relaxation in QDs can occur by various mechanisms. For ensembles with inhomogeneous environments (e.g., static offsets in spin state energies), the slight difference in phase introduced to the photon polarization as it propagates causes dephasing labeled T2*. Dephasing of this type can be counteracted in the ensemble by the so-called “echo” methods that reverse the phase evolution with appropriately timed pulses. This reveals the intrinsic dephasing time T2 that is dictated by the aforementioned fluctuations in spin-state energies due to various irreversible electron–hole or bath interactions. For example, spin–lattice interactions that cause small fluctuations in the electrostatic environment commonly reduce the coherence time with a characteristic temperature dependence.^74^ Exciton decoherence can be accelerated through pure dephasing or phonon-assisted population decay, and the various forms of exciton–phonon coupling in QDs have been previously reviewed.^75^ Colloidal QDs have the additional consideration of vibrations from ligands that may couple to excitons.^76^ Isolating the QD from its neighbors and reducing excitation density ameliorates exciton–exciton coupling issues that might also cause dephasing. The nature of many excitonic semiconductor QDs is that they are made from heavy atoms that also carry nuclear spin moments that can interact with exciton spins via hyperfine effects. As with the field of single-electron transistors for quantum applications, highly enriched (i.e., nuclear spin-free) materials made of lighter atoms (e.g., Si) are preferred to increase coherence times.^77^ However, these systems often do not possess a direct band gap or are amenable to quantum confinement, and this is an area that deserves additional research. In principle, if the interactions are well-defined spatially, the nuclear spin can act as a quantum register (e.g., InAs). The use of a ground-state species as the initialized spin circumvents the often short lifetime of excited states in QDs and QWs.

Whereas the exciton fine structure of spherical zincblende or wurtzite QDs forms the basis of many foundational studies in this field, other shapes and crystal symmetries can lead to different outcomes. For example, the prolate or oblate distortion of a spherical QD, primarily due to strain, can alter the energetic ordering of fine-structures states, changing the fine structure relaxation dynamics,^78^ as can intentionally fabricating lower symmetry nanostructures.^79^ In addition, making nanoheterostructures designed to specifically separate electron and hole can have advantages in terms of elongating spin coherence,^80^ in analogy with the TMDC/molecule heterobilayers mentioned below. Intriguingly, certain perovskite nanocrystals possess a “bright” ground-state triplet exciton,^81^ which is opposite to what is known for zincblende nanocrystals and could form the basis of interesting exciton spin polarization effects.^82^

## 3 Experimental Techniques for Probing Spin Alignment and its Decay

The manifestations of photoinduced spin alignment and the various time scales and mechanisms of its decay are best revealed through incisive experimental probes. Common to the experiments are pulsed laser excitation with controlled polarization of the beams. Important elements include analysis of the time-dependent change in polarization as a probe beam traverses the sample. The manner in which this is achieved is correlated with the type of information desired and sensitivity required, within constraints of experimental complexity. Time-resolved circular dichroism (TRCD) is arguably the simplest, as in its basic form it does not require polarization analysis of the probe. Time-resolved Faraday rotation (TRFR) and magneto-optical Kerr effect (MOKE) both utilize an applied magnetic field to induce macroscopic spin alignment that is probed by rotation of the linear polarization, differing primarily by the beam geometry. Cross-polarized transient grating (CPTG) spectroscopy employs the interference of induced sample polarizations to monitor the relaxation of spin alignment in a background-free geometry.

### 3.1 Faraday Rotation

**Figure 7:**
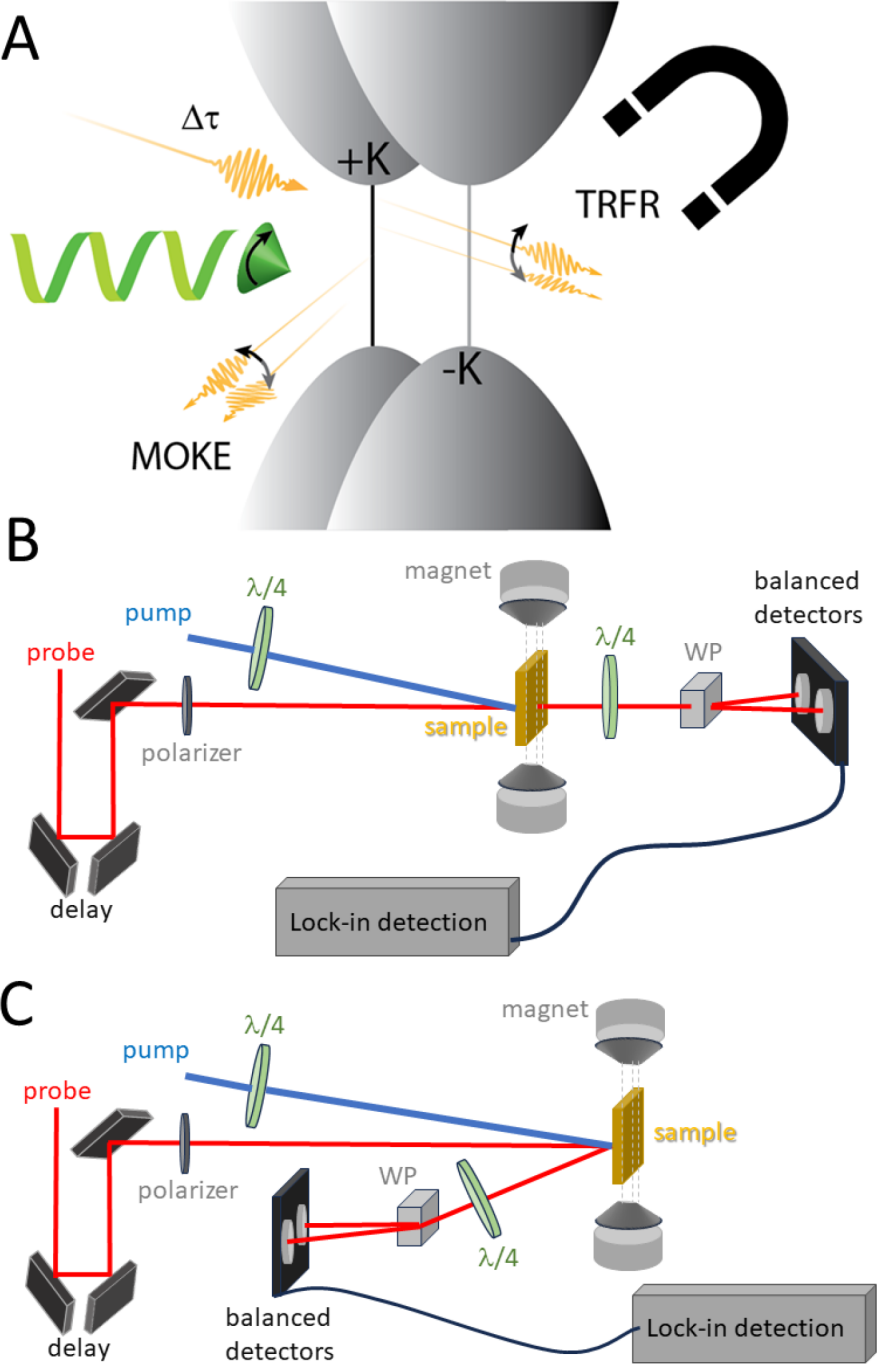
A, Schematic of the experimental time-resolved Faraday rotation (TRFR) and magneto-optical Kerr effect (MOKE), where the linearly polarized probe beam (yellow arrow) is rotated by the sample, depending on the interaction with the exciton (gray or black arrows). Transmission (TRFR) and reflection (MOKE) of the rotated polarization angle are collected as a function of delay time from the circularly polarized pump beam (green). B, Beam diagram showing optical path for TRFR. WP = Wollaston prism for separating orthogonal polarizations that become unbalanced upon rotation due to interaction with magnetic dipoles in the sample. C, Beam diagram showing optical path for MOKE.

One of the established techniques for monitoring spin coherence on ultrafast time scales is time-resolved Faraday rotation (TRFR). Its use in QD and QW systems is particularly salient, as many samples exhibit inhomogeneous broadening, which degrades persistent coherence on a picosecond time scale, and thus short pulses are required. A circularly polarized pump pulse is used to photoexcite a sample to which a magnetic field is also applied, either parallel (Faraday geometry) or perpendicular (Voigt geometry) to the propagating beam direction. A linearly polarized probe pulse interrogates the sample at a delay time τ, and its polarization is analyzed after traversing the sample, typically using a differential detection scheme for high sensitivity to small degrees of polarization rotation. The rotation of the linear polarization is caused by a birefringence for right-vs left-circularly polarized light that is proportional to the projection of the exciton spin along the axis of the excitation polarization, sometimes referred to as spin magnetization. Applying the Faraday geometry leads to measurement of the T1, or the longitudinal spin relaxation time, compared with the measurement of T2*(transverse relaxation) in the Voigt geometry. The degree to which these geometries separately measure homogeneous vs inhomogeneous effects depends on the net alignment of the sample. In the Voigt geometry, the rotation also varies with time sinusoidally due to Larmor spin precession, and this oscillation is damped by spin relaxation away from the initial spin orientation. The Larmor frequency is proportional to *gmBH*/*h*, and the analysis of resulting frequencies can reveal g-factors associated with particular spins, for example electrons vs excitons.^68,83^ See Figure 7.

Large magnetic fields in the range of 1–10 T are commonly applied to increase the rotation angle into an experimentally measurable range. Such high field strengths can in some cases cause altered dynamics compared with zero-field conditions due to field-induced state mixing and avoided crossings. Also, application of high field may be impractical in some situations, or inhomogeneous fields may induce spurious dephasing. Techniques that can be applied “in situ” and without perturbation to native dynamics may have broader application in sensing applications.

For TMDCs the information provided by TRFR relates to spin-valley polarization lifetimes, which generally fall in the subpicosecond to picosecond range.^84^ Heterostructures involving multiple TMDC layers, or TMDC/molecular layers have claimed advantage over neat systems. This is a result of opportunities for exciton dissociation that may avoid deleterious strong exchange interactions.^85^ Promoting charge separation while simultaneous maintaining spin polarization is a major challenge that likely requires precise interfacial design. However, many of the design principles have yet to be uncovered, partially due to difficulty in deriving the accurate structure of heterogeneous nanoscale interfaces.

Interestingly, Ouyang and Awschalom used TRFR to demonstrate the transfer of oriented exciton spins across the QD-molecule-QD interface,^86^ although the degree of spin polarization transfer was only about 0.2. Increasing this yield could enable a more versatile partnership between QDs or TMDCs and molecules, in which the beneficial properties of one complements the other. For example, QDs and TMDCs are excellent light absorbers and sources of light-induced spin states, while molecules can be tailored to transport charge or excitons^87^ in a low spin–orbit coupling environment. Molecules can also host unique spin state manifolds, such as radical pairs^88^ or triplet excitons.^89^

### 3.2 Magneto-optical Kerr Effect

The magneto-optical Kerr effect (MOKE) is analogous to the Faraday effect but considers polarized light reflected off a sample instead of transmitted through it (Figure 7).^90−92^ It has often been used to measure kinetic perturbations to magnetization (e.g., due to thermal effects^93^) in magnetic materials using a linearly polarized pump and probe. However, for nonmagnetic systems of interest here it can be operated in a similar configuration to the time-resolved Faraday rotation experimental setup, wherein a circularly polarized pump beam excites the sample while a linearly polarized beam probes it. The effect is attributed to the off-diagonal elements of the dielectric tensor resulting in an anisotropic permittivity of the material. This anisotropy induces a phase shift of the incident polarized light which can be measured as a function of delay time. The loss of pump-induced magnetization due to various processes involving electron–electron and electron–phonon interactions typically follows.

As Faraday and Kerr rotation measure the differential dielectric response to the circularly polarized light, this allows for increased sensitivity compared with typical pump–probe experiments. Polarization independent background contributions can be neglected and a 5-fold improvement in the signal-to-noise ratio (*S*/*N*) of the optical Stark effect was reported, allowing for the observation of a 4 μeV shift in MoS_2_.^94^ Similarly, the valley Stark effect was investigated in WS_2_ and WSe_2_ monolayers.^36,94^

Perturbation of the measured system with magnetic fields is a concern as mentioned above. However, a study using nonresonant Kerr rotation with an applied transverse magnetic field has demonstrated that the polarization of the electron spin of a quantum dot can be directly monitored with minimal effect on the system.^95^

### 3.3 Time-Resolved Circular Dichroism

**Figure 8:**
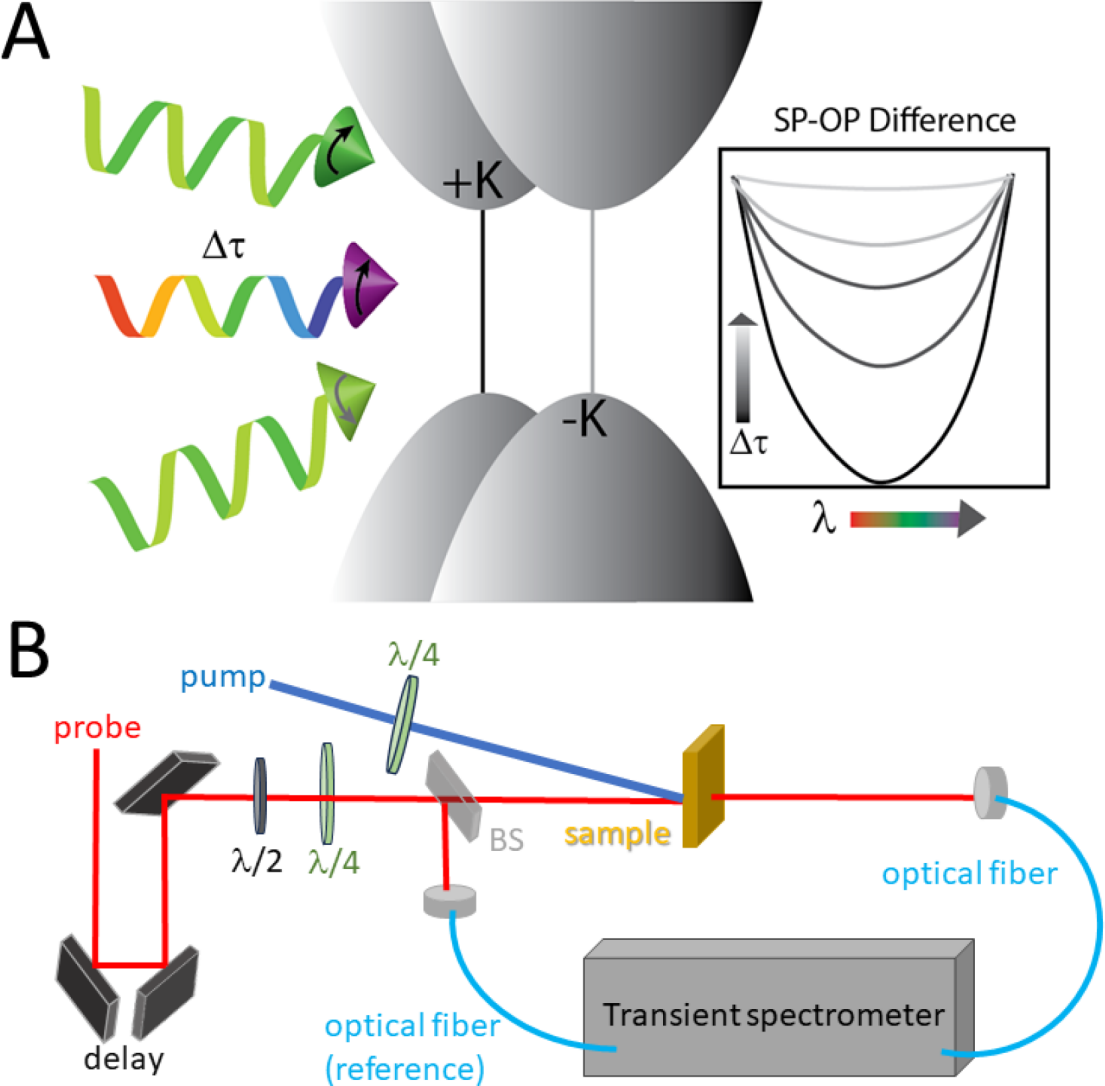
A, Schematic of the experimental time-resolved circular dichroism setup. The excitation is either right or left circularly polarized to align or antialign with the circularly polarized probe (SP or OP). The difference in photoinduced signals under these two conditions gives the TRCD signal shown in inset, emulating an exciton bleach that undergoes spin relaxation on the time scale Δ*t*. B, Beam path diagram for TRCD. BS = beamsplitter to monitor probe intensity changes for referencing the photoinduced transient spectra. In this diagram, SP and OP experiments are performed consecutively, with the TRCD signal resulting from subtraction. Fast modulation between SP and OP can be accomplished with electro-optic elements, and lock-in detection used to directly measure SP-OP at a specific wavelength.

Circular dichroism (CD) is the difference in absorption of left- and right-handed circularly polarized light, sometimes termed “optical activity” (Figure 8a). A popular implementation involves the use of a magnetic field applied to the sample to produce a net spin alignment that is then detected by the difference in absorption between right- and left-circularly polarized light, commonly termed magnetic CD or MCD. Using time-resolved circular dichroism (TRCD) spectroscopy one can instead imprint a spin orientation using the pump light helicity and the material transition dipole response to it, and then probe the produced orientation as a function of delay time to resolve the lifetime of spin-polarized states. This TRCD experimental setup employs an ultrafast pump–probe transient absorption instrument using a circularly polarized pump beam to photoexcite the sample, which is then probed with either the same circular polarization (SCP) or the opposite circular polarization (OCP), Figure 8b. The polarization is then defined as
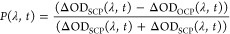
1

Using TRCD can provide substantial insights into the competing many-body mechanisms within TMDCs, namely the optical Stark effect, the direct Coulomb driven Dexter-like intervalley interaction and the intravalley exciton exchange interactions.^96^

Although it is relatively simple to implement and requires no magnetic field, TRCD is a high-background experiment, which may limit *S*/*N* compared with other measures of spin-valley relaxation. For example, if the transient signals have noise or drift during the measurement, these artifacts will map onto the TRCD signal, complicating analysis. We note that the TRCD techniques have additional elaborations that can be employed to derive more specific information. For example, probing in the X-ray range (TrXMCD) allows for probing dynamic structural elements of helicity as it relates to material’s optical activity.^97^

### 3.4 Cross-Polarized Transient Grating

**Figure 9:**
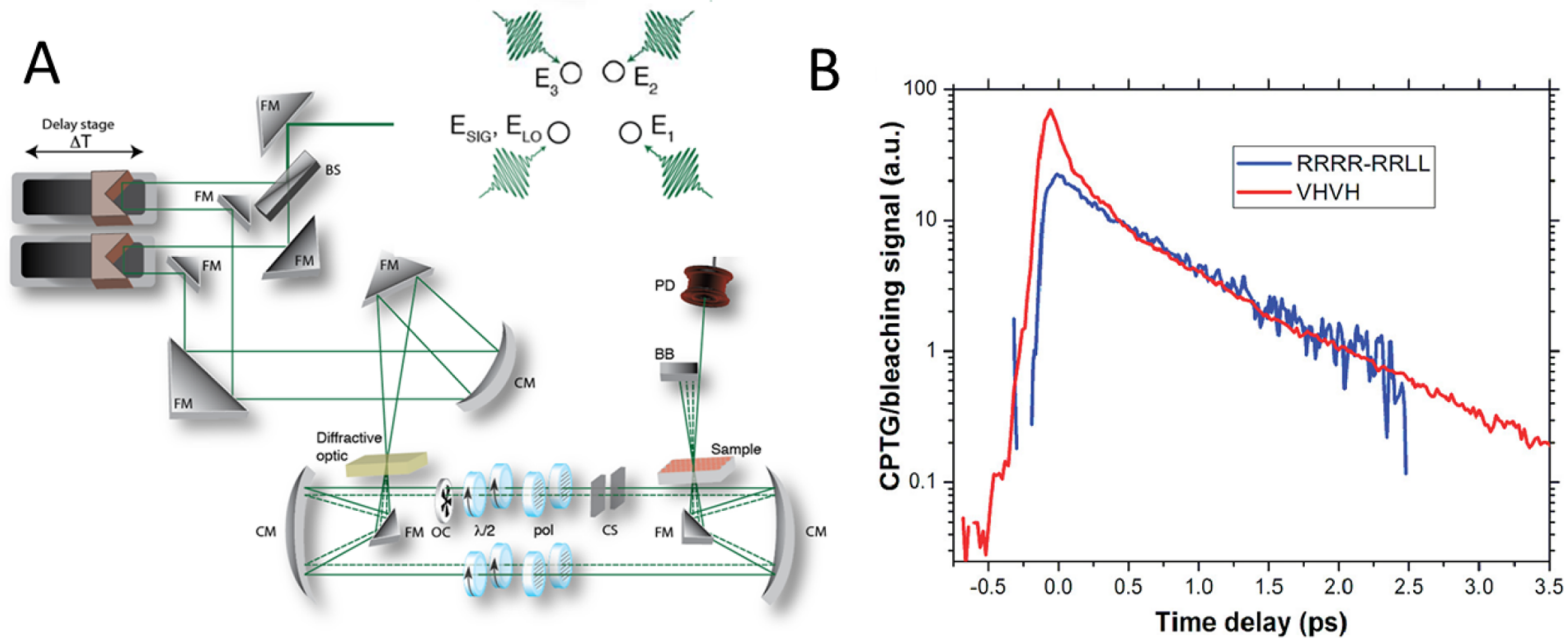
A, Schematic of the experimental cross-polarized grating setup. Four equivalent beams are generated via a transmission diffraction grating (diffractive optic) and situated in a boxcar configuration (zoom of beam configuration in upper right). Folding mirrors (FM) and curved mirrors (CM) direct the beams off delay stages and through half waveplate (λ/2)/polarizer combinations. An optical chopper (OC) modulates one beam for lock-in detection, and two coverslips (CS) provide intensity and delay control for the local oscillator (LO). Reproduced from ref (73). Copyright 2013, American Chemical Society. B, Comparison of TRCD (RRRR-RRLL) and CPTG (VHVH) signals for the exciton fine structure relaxation of an ensemble of PbS QDs. Reproduced from ref (72). Copyright 2008 American Chemical Society.

Cross-polarized transient grating (CPTG) spectroscopy relies on many of the same underlying principles as TRCD, but is implemented with a unique optical architecture. The incoming beams are arranged in a so-called “boxcar” phase-matching configuration, in which the two adjacent coincident excitation pulses have orthogonal polarization (Figure 9). The pulses, *k*_1_ and *k*_2_, interact with the QD transition moments to produce oriented excitons (e.g., *F* = ± 1). Note that this type of “spin” or “phase” grating is unlike the more common population grating that occurs for excitation pulses that have the same polarization.^98^ Even in the absence of strict alignment, individual QDs possess local exciton orientation, depending on their crystallographic alignment with the polarization light vector and its helicity. The ensemble of oriented excitons undergoes spin relaxation as described above during a waiting time τ, at which time a third pulse *k*_3_ interrogates the sample through the third-order polarization. This pulse effectively diffracts in the phase-matched direction, often accompanied by a weak fourth pulse that serves as a local oscillator for heterodyne detection. The decay of the spin grating due to spin relaxation or diffusion is followed by the loss of CPTG signal on the detector. Relaxation is often defined by spontaneous spin “flips” that destroy phase coherence, as the contribution of “flipped” and “unflipped” spins eventually become equal, and optical signals return to baseline. The unique direction of the signal means it is zero background, which can be advantageous for obtaining very high *S*/*N*. A comparison between CPTG and TRCD signals for the same sample is shown in Figure 9b.^72^ Analyzing the spin flipping rates rates as a function of QD size in terms of Fermi’s golden rule has led to estimates of the splitting of excitonic levels within the fine structure and an understanding of possible mechanisms.^99^

Although the CPTG technique has found utility for understanding spin relaxation and exciton dynamics in QDs and perovskites, it has yet to be utilized for 2D materials, although related experiments have been reported.^100^ One advantage of a grating setup is that it can provide simultaneous dynamics and diffusion information on a nm length scale.^101^ Nonlinear techniques amenable to high spatial resolution can offer an additional opportunity in the field of spin dynamics toward understanding how spin coherence depends on position in a sample, particularly with respect to edges, defects, and interfaces. Some initial studies have reported significant variations on different regions of MoS_2_.^102^ Employing these optical techniques in a microscopy mode more widely could provide useful new information into the mechanisms of spin-valley relaxation and spin transfer in heterogeneous or hybrid samples.

## 4 Manipulating Spins Optically

All-optical manipulation of a spin ensemble is a key operation for the applications discussed in this review, and various strategies have been developed to address this. The most ubiquitous method is based on a nonresonant Stark effect, wherein transitions related to a specific spin state can be shifted transiently, resulting in a pseudomagnetic field effect. For 2D magnets, the coupling between optical excitation and the magnetic state is a natural playground for control, and strategies thus far rely on altering magnetic behavior using selective spin-transfer torque and spin–orbit effects on the photogenerated spin currents. As we will discuss below, further understanding of the nature of the exciton states could enable novel approaches.

### 4.1 Optical Stark Effect

One of the primary classes of optically induced spin manipulation in nanostructures derives from the optical Stark effect (OSE).^103,104^ It has been used to demonstrate rotations of electron spin orientations in semiconductors on a picosecond time scale. While there are various experimental implementations of OSE, particularly depending on the application of an external magnetic field, the general principles remain the same. The optical Stark effect primarily arises when a sub-band gap optical pulse excites virtual exciton states, sometimes referred to as “dressed” states, which can also be considered to arise from a stimulated Raman transition. A subsequent probe pulse interacts with the dressed states generated by the nonresonant pump, resulting in a transiently shifted absorption of the lowest excitonic features due to a Stark effect: *δE* = ((μ_0_X)^2^ < *F* > ^2^)/Δ, where μ_0_*X* is the transition dipole moment between the ground and a nondegenerate exciton state, ⟨*F*⟩ is the time-averaged electric field of excitation light, and Δ is the detuning of the excitation energy from the exciton resonance (Δ = *E*_0_ – *hν*).

The pure transient optical Stark effect, absent contributions from resonant pumping and involving just a single two-level system, is expected to show a derivative-like difference signal that decays with the pulse overlap time. However, due to the presence of the transition dipole moment term in the response, the selection rules of the excitonic transition apply just as in resonant pumping, thus the helicity of pump and probe light combinations can affect the strength and shape of the transient excitonic shift signal depending on the material system. The optical stark effect in WS_2_ was shown to be tuned by as much as 18 meV,^105^ and 10 meV in WSe_2_.^106^ Selected perovskite systems show similarly large OSE, which improves separability and detectability of spin states.^107^ The large OSE is driven primarily by the large exciton transition dipole moment in these materials, which is a hallmark of TMDCs and perovskites, allowing for monolayer samples to be studied using optical spectroscopy.

**Figure 10:**
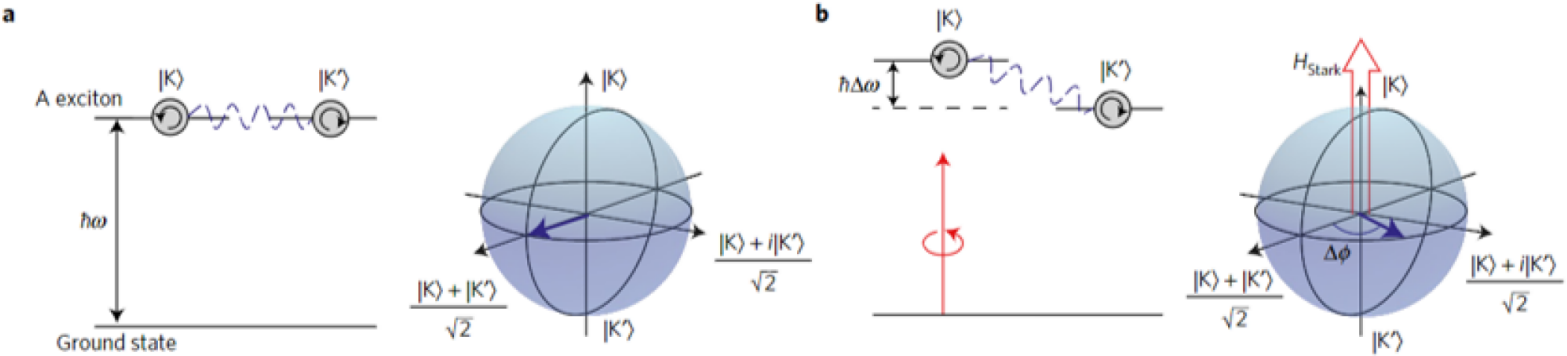
Schematic of the all-optical valley pseudospin manipulation showing (a) the pseudospin in the equatorial plane and (b) the pseudospin rotated by an angle Δϕ by applying a polarized pulse. Reproduced with permission ref (40). Copyright 2016 Springer Nature.

It has been shown that valley pseudospin can be manipulated optically by utilizing the Stark effect present in the TMDC monolayers (Figure 10). By first exciting the WSe_2_ with near-resonant linearly polarized light, the valley pseudospins are aligned on the equatorial plane of the Bloch sphere. Then a circularly polarized pulse below the band gap leads to a rotation of the pseudospin angle, Δϕ, determined by the helicity of the photoluminescence. The rotation is due to the second polarized pulse lifting the valley degeneracy by ℏΔ*ω* and introducing a dynamic phase difference Δϕ ∼ Δω Δ*t*, where the pulse has a duration Δ*t*.^40^ Optically addressing exciton spins is of value to develop quantum devices. However, although these systems are of interest, they currently have very short decoherence and exciton lifetimes.

The 2D and perovskite systems described above have an intrinsic advantage over many QD systems in terms of the magnitude of OSE effects, but nonetheless QDs have been utilized in similar schemes. Ultrafast “tipping” pulses that rely on OSE have been shown to rotate the exciton spin polarization in QD systems.^108^ When a strong external magnetic field is applied, the photogenerated spin states precess about the field axis at the Larmor frequency. Subsequent timed optical pulses act on the spin as π or π/2 pulses act in NMR or EPR. Thus, gate operations can be performed as quickly as the frequency of the precession, which is dictated by the magnetic field strength and not the tipping pulse width.

### 4.2 Ultrafast Magnetic Order Transitions

In 2D magnetic systems, ferromagnetism-induced hysteretic optical signals have been observed through photoluminescence^109^ (PL), Kerr rotation,^110^ or circular dichroism measurements. These results unveiled ferromagnetic coupling between the Cr spins within a monolayer plane with easy axis magnetization oriented out-of-plane for CrBr_3_ and CrI_3_ and in-plane for CrCl_3_. Thickness-dependent interplane ferromagnetic and antiferromagnetic coupling in CrI_3_ multilayers as well as light-mediated ferromagnetic response in doped TMDCs were also detected.^111−113^ However, these optical methods have primarily been used as magnetization probes while the interplay between the magnetic state, and the optical excitations remain unexplored. The incomplete understanding of the correlated exciton-magnetization states constitutes a bottleneck in further development of the two-dimensional ferromagnetic structures and devices. An important aspect of such interplay is the ability to optically pump the electronic spins. In CrBr_3_ and CrI_3_ the easy-axis anisotropy enables the out-of-plane magnetization direction, which *in principle* can favor particular spin alignments within the photoexcited excitonic population, leading to plausible mechanisms of controlling the spin and/or magnetization properties of the material with light, a phenomenon that Grzeszczyk et al. explore in great detail.

Manipulating magnetization more rapidly and efficiently is essential to improve on existing magnetic reading and recording technology. To tackle this problem, all-optical switching of ferro- and ferrimagnetic materials using ultrafast pulses has been investigated extensively in the last couple of decades.^114^ The initial laser pulse sets the spins into nonequilibrium states that can then be manipulated with subsequent pulses. One such mechanism relies on laser-induced spin currents that can be manipulated through spin transfer torque. The helicity dependent analogue, optical spin transfer torque, or the inverse Faraday effect induced torque can also affect the magnetization dynamics in thin films as^115−117^
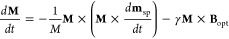
2where **M** is the vector magnetization of the ferromagnet and *M* its magnitude, γ is the gyromagnetic ratio, **m**_sp_ is the induced spin polarization, and **B**_opt_ is the effective magnetic field along the direction of light propagation.^114^ Helicity dependent all-optical switching of ferromagnetic films and nanostructures have been successfully achieved over the years.^114,118−120^ More recently, this has been explored in 2D van der Waals magnets. CrI_3_ is combined with WSe_2_, which is used to manipulate the magnetic order of the 2D ferromagnet through spin-dependent charge transfer. Complete switching of the out-of-plane magnetization was achieved with multiple femtosecond pulses, using both linear and circularly polarized light.^121^ A theoretical study has also been presented to determine the feasibility of optical switching in 2D ferrimagnetic MXenes.^122^

### 4.3 Topologically Structured Light

**Figure 11:**
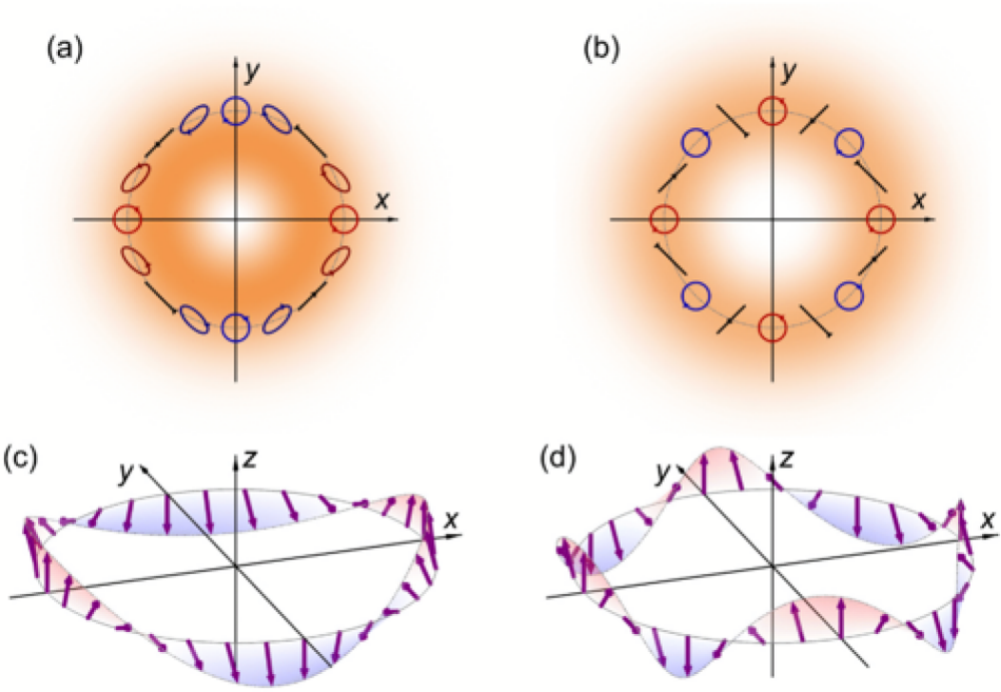
Cartoon representation of the result of vector vortex beams polarization distribution (a), (b) on the spin orientation of a semiconductor quantum well, given optical selection rules (c), (d). Different mode structures are illustrated in (a–c) vs (b–d). Reproduced with permission from ref (123). Copyright 2023 American Physical Society.

The notion of using topologically structured light to induce patterned excitonic spin polarization effects in nanomaterials is an intriguing variation to methods described above. Vector vortex beams have a spatial helicity structure that imprints its orbital angular momentum onto the photoexcited material, much in the same way that CPTG imprints angular momentum in a grating pattern through cross-polarization of coincident beams. The elaboration here is to include a helical spatial component, for example by wrapping the spin grating modulation into a circular form, Figure 11. An example involving a semiconductor quantum well was recently demonstrated, in which a persistent spin helix was formed due to balancing of spin–orbit interactions in GaAs/AlGaAs. Effective magnetic fields due to spin–orbit coupling act on diffusing spins and cause the photoexcited spin distribution to evolve toward unique patterns at later times.^123^ The many parameters available in the vector vortex beam allow for a highly tailorable spin distribution that could be employed in potentially useful spintronic schemes.

## 5 Outlook and Opportunities

### 5.1 Symmetry Lowering

The aforementioned analysis of exciton wave functions has important consequences in fundamental understandings of excitons. For example, in the many-body literature of “Frenkel” excitons they are often characterized as d-d transitions that are dipole forbidden, a principle that is popularly known as the Laporte rule. However, in reality, the Laporte rule relaxes because a part of the excitonic wave function resides on the *p* states of the ligands. Through pie charts of the exciton wave functions, Acharya et al.^24^ show the quantitative distribution of the electron and hole parts of the wave functions on different intra- and intersite channels. While the intrasite *dd* transitions are indeed forbidden, the intersite *dd* transitions are dipole allowed. This is a physical principle that is often not fully appreciated. Acharya et al.^24^ show the exact quantitative enhancements in such intersite components to the exciton wave functions going from CrBr_3_ to CrI_3_ that make the ground state excitons intrinsically brighter in CrI_3_. Often the literature concerning such strongly bound excitons confuses two important factors: what gives rise to the excitons and what makes them bright. They show that while the fundamental origin of these excitons are *t*_2*g*_ → *e*_*g*_ transitions, which is sufficiently captured in a many body perturbative approach like QSGW*^*, the excitons become brighter due to symmetry lowering mechanisms like, *dp* hybridization, spin–orbit coupling and excitons coupling with odd-parity phonons.^24^ Such quantitative analysis of the exciton wave functions provides the desired information on the tunability of the “Frenkel” excitons in real materials. For example, CrI_3_ excitons being composed of more dipole-like components compared to CrBr_3_ should be more tunable via a change in substrate or strain. Further, they analyze the excitons in the band basis and show that a host of bands residing over several electron volts and electrons and holes residing at all **q** points in all such bands contribute to the exciton formation.^24^ This effectively makes a conventional “mass” analysis of the electrons and holes that take part in the exciton formation untenable.

**Figure 12:**
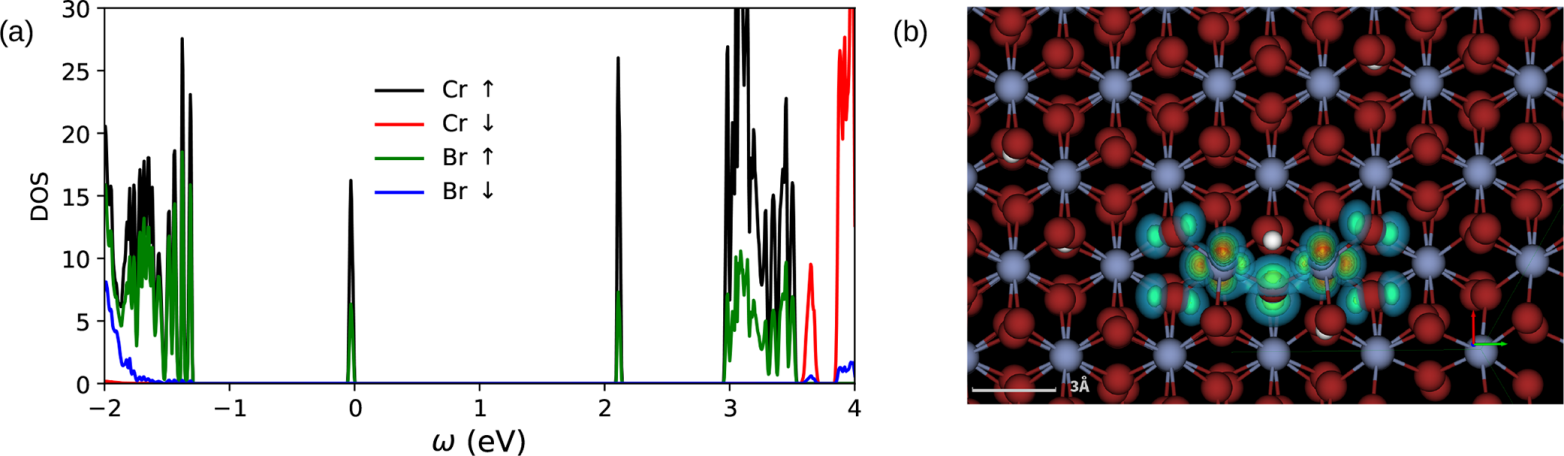
(a) Electronic spin-projected density of states (DOS) are shown for CrBr_3_ in the presence of Br-vacancy. The nondispersive vacancy states are mostly Cr-*d* like with moderate contribution from Br-*p*. Adapted with permission from ref (57). CC BY 4.0 DEED. (b) Vacancy lowers the symmetry of the underlying crystal and makes the Frenkel exciton anisotropic. The exciton spectral weight shifts anisotropically to the Cr atoms closer to the location of the vacancy (shown in a white dot). Red and blue colors represent the highest and the lowest spectral weights, respectively.

Recent work also puts the focus back on symmetry lowering mechanisms that can tune excitons in these 2D magnets.^24,56,57^ For example, in ref (57) the authors show how introduction of a single halogen vacancy lowers the symmetry of the exciton state, making it brighter. They also observe magnetically split nondispersive vacancy states which could be useful in realizing single-photon emitters with neutral charge and spin degree of freedom and for qubits. We believe a similar “brightening” mechanism can be realized through introduction of strain, different substrates, or making heterostructures of these magnets with nonmagnetic TMDCs. A single charged Br vacancy was created in a supercell of CrBr_3_ with 72 atoms (54 Br and 18 Cr). In the presence of a vacancy, single particle calculations were performed on a 8 × 8 × 1 *k*-mesh while the dynamical self-energy Σ(*k*) was constructed using a 4 × 4 × 1 *k*-mesh. The size of the two-particle Hamiltonian that we diagonalized was 4 × 4 × 96 × 56 (*n*_*k*_× *n*_*k*_ × *N*_*v*_ × *N*_*c*_), i.e., 86 016. Within our QSGW approach, a ∼2.1 eV splitting was observed between the occupied and unoccupied vacancy states. These states are entirely dispersionless and, hence, do not interact with each other. The primary orbital character of these vacancy states is shown in Figure 12. As the degeneracy is lifted the deepest lying excitonic eigenvalue at 1.3 eV splits into two eigenvalues at 1.2 and 1.37 eV. Lowered symmetry makes the exciton anisotropic (see Figure 12) and enhances the oscillator strength of the ground state exciton by at least 2 orders of magnitude. Another recent study activates the dark excitons in CrCl_3_ by forming heterostructures with WSe_2_.^124^ Moreover, additional studies^22−24^ show that the direct or indirect nature of the electronic band gaps do change going from bulk to single layer. This can have important consequences in tuning the brightness of the excitons across different numbers of layers. Importantly enough, this stresses the fact that a precise understanding of the excitons and their oscillator strengths is extremely involved, and the usual oversimplification of the problem as physics in single layers that are only weakly coupled between different layers often does not aid our understanding. Layer dependent changes in lattice dimensionality and surface to bulk ratio lead to crucial changes in electronic screening that changes the relative alignments and potentials of *d* and *p* states and can lead to qualitative changes in both electronic and excitonic wave functions.

Furthering the discussions on symmetry, over the past few years a new group of 2D magnets have been realized, although only a few of them as of now, in a puckered orthorhombic crystal structure.^125−127^ Such anisotropic 2D magnets have highly directional excitons, with the additional property that their spin components get locked in the plane. These derive from the FeOCl structure prototype with *Pmmn* space group, including, e.g., CrBrS and CrOCl. We predict that the exciton-magnetization coupling in these systems will be highly tunable wherein some excited states of the excitons orient perpendicularly to the ground state, thus allowing for a polarization-selective drive of exciton orientation and the accompanying spin in a three-level system. Such cross-polarized exciton spin composites can be accessed via polarized near-infrared spectroscopy, which is compatible with low-loss telecommunications platforms, and can be manipulated with frequencies in the THz without applied magnetic field typically required for two-level quantum systems. Puckered magnets like CrBrS also come in the same crystal structure as the puckered nonmagnetic semiconductor with a small band gap, black phosphorus. The starkly different electronic and excitonic properties of these different classes of systems with similar crystalline symmetry promises highly tunable heterostructural properties.

### 5.2 Magnetic Order–Disorder Transition

All magnets go through a magnetic order–disorder transition at some finite temperatures. The *T*_c,N_ depends^128^ sensitively on the effective spin dimensionality and number of layers of the magnetic systems. Most theoretical and experimental approaches focus on the ordered ground state while the nature of the magnetic disordering can provide useful insights. A recent combined ARPES and QSGW*^* study^129^ successfully describes the electronic structure in CrBrS in the high temperature (>146 K) magnetically disordered (paramagnetic) phase. Monolayers of these puckered magnets are ferromagnetic while they are antiferromagnetic in bilayer and bulk variants. This suggests that excitonic wave functions, their spin components, and oscillator strengths could be significantly dependent on the number of layers. Also, the impact of the magnetic order–disorder transition on excitons is not very well understood. In a recent work, Ruta et al.^130^ shows that the oscillator strength of the ground state excitons in bulk CrBrS enhances significantly due to magnetic ordering. Their combined theory and experimental work shows that the quantitative nature of the enhancement can be described by QSGW*^* theory where magnetic ordering favors the electron part of the excitonic wave function to delocalize and reduce onsite *d-d* contributions to the exciton spectral weight, leading to enhancement in excitonic oscillator strength. The building blocks for bulk CrBrS are ferromagnetic layers with weak antiferromagnetic vdW coupling. In such ferromagnetic monolayers, electron hopping is favored between atoms of like spin. Hence, magnetic disordering should reduce the itineracy of the electrons and reduce kinetic energies of the electronic bands. Ruta et al. shows that this is indeed the case within their QSGW*^* theory, and the electron part of the exciton wave function gains a significant amount of kinetic energy through magnetic ordering. While this principle is correct for systems with ferromagnetic chains,^131−133^ this is not true universally. Acharya et al.^134^ shows that magnetic disordering significantly enhances oscillator strength of the excitons in bulk AFM insulators like NiO and MnF_2_, and in such cases the exact nature of the electronic hopping that contributes to the exciton spectral weight is less trivial in nature compared to the aforementioned scenario of FM chains.

### 5.3 Chirality

**Figure 13:**
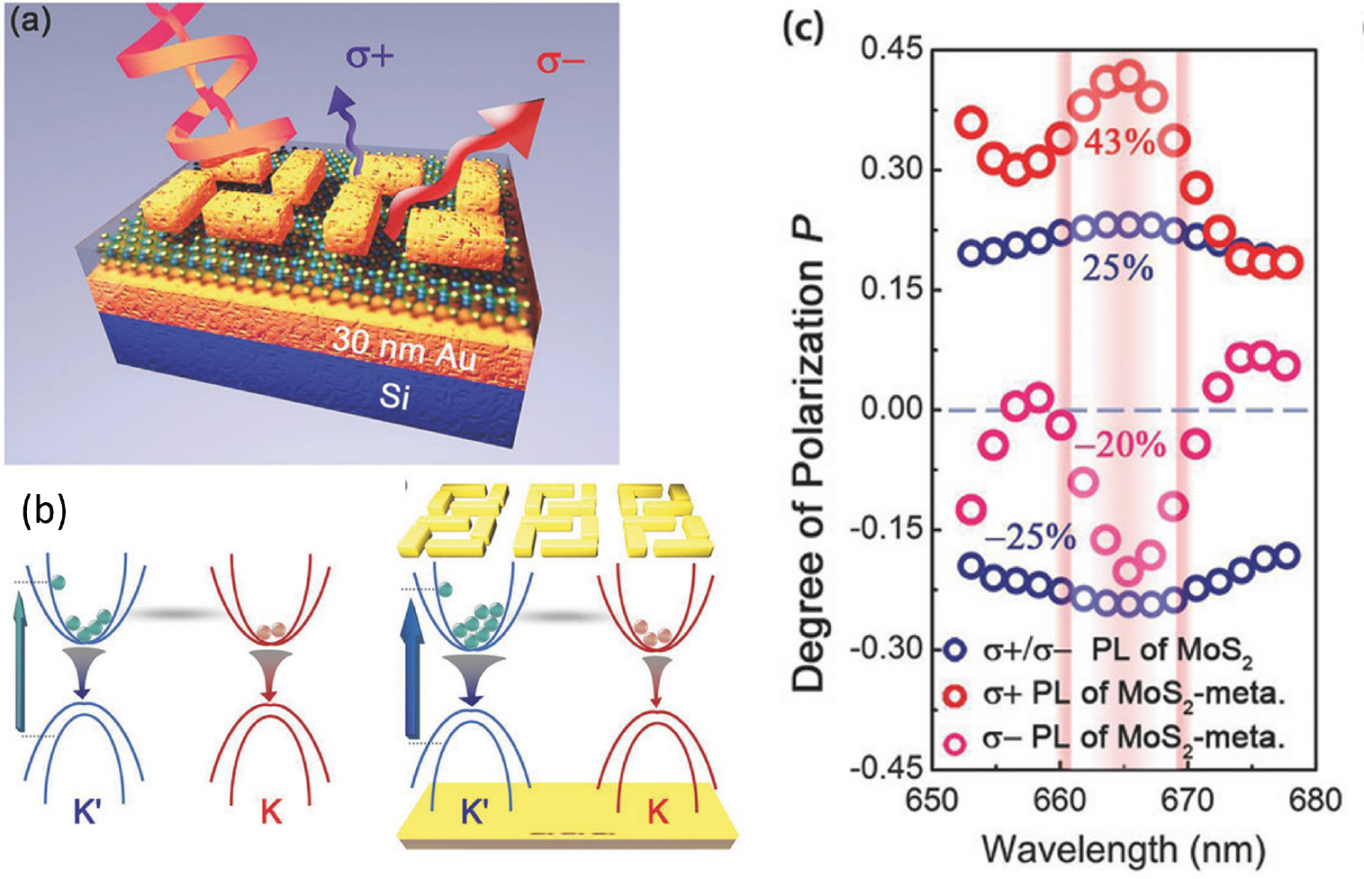
(a) Schematic of MoS_2_ metasurface structure consisting of a Si substrate with Au, MoS_2_ and the chiral metasurface deposited on top. (b) Schematics of the MoS_2_ monolayer (left) and MoS_2_ metasurface (right) energy bands. (c) Distribution of the degree of valley polarization, achiral for Mo*S*_2_ and chiral for the MoS_2_-metasurface. Adapted with permission from ref (135). Copyright 2018 WILEY-VCH.

The notion of imprinting chirality onto a material using a helical light field discussed above can also be accomplished using a proximity effect from a nearby chiral layer. In one example, a chiral metasurface composed of plasmonic structures was grown proximal to a single layer of MoS_2_, Figure 13.^135^ The plasmonic material responds to helical light, and the generated chirality enhanced valley polarization differences in MoS_2_, as measured by CPL. Even linear polarization impingent on the nanostructures transduces a valley polarization difference in MoS_2_. Similary, enhanced valley contrast was reported by depositing chiral molecules directly on MoS_2_.^136^ Other recent advances suggest that some of the polarization selectivity of charge transfer upon photoexcitation is instrinsic to the chiral nature of the molecules used in the perovskite structure, and not necessarily created by the light source.^137^ Although this general phenomenon of chirality induced spin selectivity (CISS) has promise in fields adjacent to the subject of this review,^138^ for purposes of fast optical modulation of spin behavior and associated magnetism the relatively fixed nature of chiral sense makes these architectures less relevant.

### 5.4 Exciton–Phonon Coupling

Another crucial aspect of the exciton physics is the coupling of excitons and their spins with the lattice. Exciton–phonon coupling has been studied in recent years in various 2D systems.^139−150^ Diffusion and relaxation dynamics of excitons are crucially sensitive to the nature and strength of the exciton–phonon coupling, and ab initio approaches for such physical phenomena are far from fully developed. Some progress has been made on the GW front^151^ in recent years but is still far from complete. For single-component 2D TMDCs, population relaxation typically dictates the homogeneous line width, rather than pure dephasing processes associated with exciton–phonon interactions. Nevertheless, exciton–phonon coupling can modify the nature of exciton states, particularly for heterobilayers, an important area of research. Recent theoretical work has suggested that initially excited bright excitons in WSe_2_ quickly transform into delocalized interlayer excitons with momentum-indirect and spin-dark exciton character through the involvement of phonons, effectively inciting decoherence in a similar fashion to intervalley exchange processes.^152^ As with the 2D magnets, accurate computational models of exciton–phonon coupling may help to uncover the mechanisms by which spin-valley phenomena evolve on ultrafast time scales. Recent works on 2D magnets explore the coupling of excitons and their orbital and spin angular momenta^153−155^ with the lattice. A particularly interesting class that is emerging in the past few years for such phenomena is MPX_3_ (M = transition metal elements and X = S,Se). Several recent studies^156−158^ explore the strong spin- and orbital-coupling of excitons with the lattice in these classes of materials, which leads to THz ultrafast laser-induced dynamics of mutually correlated spins and lattice. It has been shown that a femtosecond laser pulse can act as an ultrafast heater and can lead to simultaneous melting of the antiferromagnetic order and excitation of selective B_*g*_ modes^159^ in these classes of systems. This is emerging as an extremely promising direction for next generation data storage and computation based on spin–lattice coupling. However, an understanding of the physical phenomena at play is still far from complete.

## 6 Applications

Applications in spintronics are the most likely to emerge from the materials and phenomena described here. The manipulation and detection of the collective properties of spin states on an ultrafast time scale fits with the goal of supplanting conventional microelectronic computing schemes, in terms of speed, reliability, and energy footprint.^160^ In situations where local magnetic switching is a write or read strategy for nonvolatile memory, optical initiation of mechanisms related to spin–orbit or spin–transfer torque (induced by current in conventional schemes) could provide unique opportunities. Terahertz generation of spin current is already considered as a method for moving beyond GHz clock rates.^161^ Pulsed optical generation of such fields that employ excitonic phenomena could provide more selective operations that leverage their unique electronic structure, and can also be further localized (due to wavelength dependence of the diffraction limit), leaving opportunities for control of memory at extremely high density and operations at high speed. Challenges in this very new space include demonstrating high fidelity for the requisite transformation (e.g., photon-to-spin-to-memory/readout), which requires better understanding of the fundamental mechanisms at play in these processes.

Spin systems that are quickly adaptable based on a weak perturbation field will find application in many contexts. Quantum sensing provides a natural outlet for such materials, as the perturbation to the spin entanglement may come from the local environment. Detailed understanding of how a particular response (e.g., circularly polarized PL) varies based on local field strength and direction can effectively calibrate a given spin system toward measurement of an unknown quantity. Nanostructures that emit light efficiently (i.e., quantum-confined excitonic systems) are particularly attractive, as they can be embedded locally yet stimulated and probed remotely. The essential elements for a quantum sensing material detailed by Degen et al. can be summarized as the initialization, control and readout stages.^162^ Due to the 2D nature of TMDCs, the electronic states are highly sensitive to their environment. This is an advantage for potential sensing applications, however it also makes them prone to decoherence. Optical manipulation of TMDCs is of interest due to the strong OSE observed in these systems. Nonetheless, the short decoherence times are severely limiting at this stage for direct applications in quantum devices. Another interesting feature that could be harnessed is the spin-valley coupling in TMDCs that correlates the spin polarization to the helicity of the photoluminescence. Although not directly applicable to quantum sensing, this mechanism could be a candidate for an optical readout mechanism. However, challenges here will be to transfer the spin-polarization of the sensing material to the 2D readout layer without perturbing the 2D material to retain the optical selectivity. QDs, however, are versatile building blocks for photon or spin qubits, as they can be constructed from a wide variety of semiconductors and have the additional lever of shape-modified exciton fine structure. They have tunable optical properties and can be integrated in photonic cavities. Moreover, the scalability of QDs toward customized ensembles with many interacting components makes them an interesting candidate for quantum information processes. Nonetheless, a critical issue that remains is their short coherence times limit their direct implementation into quantum systems.

Stimuli-enabled synaptic events are at the heart of many neuromorphic computing architectures.^163^ A change in the local optical, electrical or magnetic response that is stimulated by an external source, such as a laser with a controlled polarization, could serve as the basis for plasticity, given the right material properties. Many of the 0D and especially 2D materials discussed herein are amenable to the types of circuitry often found in neuromorphic architectures, and as demonstrated above, simultaneously possess the ability to undergo effective transitions in optomagnetic behavior through fast perturbations. In particular, neuromorphic schemes that rely on nonlinear spin-wave interference could benefit from the versatile imprinting that can be performed on materials with excitonic spin orientation.^164^ Grating and vector vortex techniques discussed above may be particularly relevant for effectively “writing” patterns of varying magnetic moments down to the nm scale, leading to elements that can be generated at high density. Further, these spin-waves can have multiple methods for responding to stimuli–excitation power via nonlinear responses, and wavelength via the different character of distinct excited states. The highly nonlinear optical response of excitonic species in nanomaterials provides a pathway for the function of such a network at reasonable light intensities. The nanoscale nature of the elements may also add value for purely photonic or optoelectronic applications, where wavelength-dependent nonlinearities are important in terms of wave mixing, power limiting, and polarization control.^165^

## 7 Conclusions

The ability to stimulate and control exciton spin orientation in nanomaterials represents a unique opportunity in many emergent fields. The key toward bridging the gap between aspiration and application is the feedback loop between fundamental understanding of the optical and electronic properties and materials design. Appropriate theoretical models and computational methods must be applied to avoid misleading conclusions. Meanwhile, selection and application of the most suitable experimental technique to probe exciton spin dynamics and manipulate its evolution are crucial. As the verisimilitude of modeling approaches real materials and their dynamical time scales, we envision new and trenchant insight that will enable advances in applications.
